# Barriers to routine G6PD testing prior to treatment with primaquine

**DOI:** 10.1186/s12936-017-1981-y

**Published:** 2017-08-10

**Authors:** Benedikt Ley, Kamala Thriemer, Jessica Jaswal, Eugenie Poirot, Mohammad Shafiul Alam, Ching Swe Phru, Wasif Ali Khan, Lek Dysoley, Gao Qi, Chong Chee Kheong, Ummi Kalthom Shamsudin, Ingrid Chen, Jimee Hwang, Roly Gosling, Ric N. Price

**Affiliations:** 1 Global and Tropical Health Division, Menzies School of Health Research, Charles Darwin University, PO Box 41096, Casuarina, Darwin, NT 0811 Australia; 2 Malaria Elimination Initiative, Global Health Group, University of California, San Francisco, CA USA; 3 Infectious Diseases Division, International Centre for Diarrhoeal Diseases Research, Bangladesh, Mohakhali, Dhaka, 1212 Bangladesh; 4 Ministry of Health, National Center for Parasitology Entomology and Malaria Control (CNM), Phnom Penh, Cambodia; 5 School of Public Health, National Institute of Public Health, Phnom Penh, Cambodia; 6 National Key Laboratory ON Parasitic Diseases, Jiangsu Institute of Parasitic Diseases, Wuxi, China; 7 Disease Control Division, Ministry of Health, Kuala Lumpur, Malaysia; 8 Division of Parasitic Diseases and Malaria, US President’s Malaria Initiative, Malaria Branch, US Centers for Disease Control and Prevention, Atlanta, GA USA; 9 Centre for Tropical Medicine and Global Health, Nuffield Department of Clinical Medicine, University of Oxford, Oxford, UK

## Abstract

**Background:**

Primaquine is essential for the radical cure of vivax malaria, however its broad application is hindered by the risk of drug-induced haemolysis in individuals with glucose-6-phosphate-dehydrogenase (G6PD) deficiency. Rapid diagnostic tests capable of diagnosing G6PD deficiency are now available, but these are not used widely.

**Methods:**

A series of qualitative interviews were conducted with policy makers and healthcare providers in four vivax-endemic countries. Routine G6PD testing is not part of current policy in Bangladesh, Cambodia or China, but it is in Malaysia. The interviews were analysed with regard to respondents perceptions of vivax malaria, -primaquine based treatment for malaria and the complexities of G6PD deficiency.

**Results:**

Three barriers to the roll-out of routine G6PD testing were identified in all sites: (a) a perceived low risk of drug-induced haemolysis; (b) the perception that vivax malaria was benign and accordingly treatment with primaquine was not regarded as a priority; and, (c) the additional costs of introducing routine testing. In Malaysia, respondents considered the current test and treat algorithm suitable and the need for an alternative approach was only considered relevant in highly mobile and hard to reach populations.

**Conclusions:**

Greater efforts are needed to increase awareness of the benefits of the radical cure of *Plasmodium vivax* and this should be supported by economic analyses exploring the cost effectiveness of routine G6PD testing.

**Electronic supplementary material:**

The online version of this article (doi:10.1186/s12936-017-1981-y) contains supplementary material, which is available to authorized users.

## Background

Almost three billion people are at risk for *Plasmodium vivax* infection globally [1, 2] and outside of Africa it is the predominant cause of malaria [3]. Although vivax malaria was once considered a benign infection, recent evidence suggests a considerable morbidity and mortality, associated mainly with anaemia due to recurrent episodes [4–6]. The propensity for *P. vivax* to relapse after prolonged periods of asymptomatic carriage undermines the patients and healthcare providers’ perceptions of the importance of radical cure, and this in turn confounds malaria control and elimination efforts.

Complete eradication of *P. vivax* will not be feasible without systematic treatment of the dormant liver forms (hypnozoites) of the parasite [7]. The only currently available treatment to kill hypnozoites is primaquine (PQ), an 8-aminoquinoline, which causes dose-dependent haemolysis in patients with inherited deficiency of the glucose-6-phosphate-dehyrdrogenase enzyme (G6PD) [8]. G6PD deficiency is the most common enzymopathy worldwide, affecting approximately 400 million people, the majority of whom are at risk for malaria [9]. To achieve effective radical cure, a sufficient total dose of PQ needs to be administered over a prolonged course, which is usually 14 days for G6PD-normal patients and weekly for 8 weeks for G6PD-deficient patients [10]. The WHO anti-malarial treatment guidelines were revised recently to recommend that wherever possible routine testing for G6PD deficiency should be undertaken prior to PQ-based radical cure. Implementing routine testing for G6PD deficiency is challenging and the WHO guidelines also state that if testing is not available, an individual risk–benefit assessment should guide the decision whether to administer without testing or withhold PQ altogether [10].

Nearly all malaria-endemic countries in the Asia-Pacific region have committed to malaria elimination by 2030; however most of these countries do not test for G6PD deficiency on a routine basis and PQ is often not prescribed [11, 12]. The inclusion of primaquine into national treatment guidelines and its implementation will be essential to the successful elimination of malaria [7], G6PD testing is currently available in a number of formats [13, 14]. The gold standard test is by spectrophotometry and flow cytometry. Although these tests quantify enzyme activity, they are expensive and require a well-functioning laboratory infrastructure. The most widely used qualitative test is the fluorescent spot test (FST) [15], which also requires laboratory infrastructure but comes at a considerably lower price and is easier to use. Despite its wide distribution the FST is not suited for remote locations without laboratory facilities and requires experience in its interpretation. A number of new lateral flow rapid diagnostic tests (RDT) for G6PD testing have been developed to overcome the challenges of the FST [16].

Primaquine radical cure has the potential to transform, the control and elimination of vivax malaria but its safe delivery will require building appropriate capacity to test G6PD status. The objective of this study was to assess key barriers to the introduction of routine G6PD testing through structured interviews with policy makers and healthcare providers in Bangladesh, Cambodia, China, and Malaysia.

## Methods

Semi-structured interviews were conducted with key policy makers and healthcare personnel; all respondents were identified based on convenience sampling. Policy makers were defined as individuals who were actively involved in generating and updating national malaria treatment guidelines and health care providers were defined as individuals who applied the national malaria treatment guidelines to the clinical management of patients. Selection of policy makers was based on their involvement in malaria-related policy decisions and healthcare providers were selected based on their work experience.

### Study sites

Sites were selected to reflect the variation in locations across Asia including a range of treatment policies. The study was conducted in four countries: Bangladesh, Cambodia, China, and Malaysia. The treatment guidelines for patients with vivax malaria in Bangladesh recommend 3 days of chloroquine (CQ) for *P. vivax* mono-infections, followed by 14 days of PQ (0.25 mg/kg) without prior G6PD testing. In Cambodia, national guidelines recommend a unified treatment policy for all species using DHA–Piperaquine (DHA–PPQ) as schizontocidal treatment with PQ only prescribed after G6PD testing [17]. In China, CQ and 8 days of PQ (0.75 mg/kg) is recommended without prior G6PD testing [11] and in Malaysia all malaria patients are admitted to hospital, G6PD testing is routine, and patients are treated with CQ and, if G6PD normal, with 14 days of PQ (0.5 mg/kg) (Table 1) [11, 18].

**Table 1:** Treatment guidelines for *Plasmodium vivax* in study sites

	Schizontocidal treatment	Hypnozoidal treatment	G6PD test recommended before PQ treatment
Bangladesh [21]	CQ	PQ 14 days (0.25 mg/kg/day)	No
China [11]	CQ	PQ 8 days (0.75 mg/kg/day)	No
Cambodia [17]	DHA–PPQ	PQ 14 days (0.25 mg/kg/day)	Yes
Malaysia [18]	CQ	PQ 14 days (0.5 mg/kg/day)	Yes

### Instrument development

Guides for the interviews were developed based on discussions between national malaria program officers, malaria researchers and healthcare providers [19]. The interviews for policy makers included questions on policy, operational, regulatory, financial, and managerial aspects of G6PD acceptability and testing. Interviews with healthcare providers provided additional perspectives from the clinical environments. The focus of these interviews was to assess the practical application and feasibility of G6PD testing, its roll-out and corresponding barriers (see Additional files 1, 2).

### Data collection

Interviews were conducted in person in Bangladesh and Cambodia by local investigators familiar with the topic, whereas in China interviews were conducted by phone. In Malaysia, interviews were conducted in person by one of the US investigators with the support of local investigators. All interviews were audio-taped, and field notes collected and written up on the day of the interview. Interviews were either conducted in the local language by local investigators and translated into English for analysis (Bangladesh, Cambodia, China) or conducted in English (Malaysia). Summaries of findings were prepared and sent to each respondent for their feedback on documented material.

### Data analyses

All interviews were transcribed and whenever not conducted in English translated verbatim into English and coded using Nvivo version 11 (QSR International, Australia). All transcripts were analysed by two researchers independently and the analyses compared. Transcripts were coded for knowledge and perceptions of vivax malaria, as well as for treatment practices and challenges associated with treatment. Additional categories were developed to capture the current practice of G6PD testing, perceived usefulness of applied tests, acceptable price as well as for perceived knowledge gaps in regards to G6PD deficiency and its diagnosis. Passages of text were read and codes assigned based on themes, and examined for frequency, trends, similarities, and differences.

When necessary direct speech in the results section was corrected for grammatical errors to improve readability; words added by investigators to improve clarity were marked by hard brackets and omissions were marked by three consecutive dots. The addition and omission of words were discussed by investigators analysing the data and were only retained if all agreed that the suggested change did not alter the sense of the respective sentence.

### Research ethics

Verbal informed consent was obtained from all respondents and participants received a translated information sheet containing details about the study, including its procedures to withdraw and measures to maintain confidentiality. The protocol was approved by the Human Research Ethics Committee of the Northern Territory Department of Health and Menzies School of Health Research, Australia (HREC 14.2312), the ethical committee of the University of San Francisco, California, USA (IRB# 14-15301) and the local ethical committees or comparable, responsible authorities from each participating country (International Centre for Diarrhoeal Research, Bangladesh (icddr,b); National Center for Parasitology Entomology and Malaria Control (CNM); Jiangsu Institute of Parasitic Diseases, China; Disease Control Division, Ministry of Health, Malaysia).

## Results

Between May 2015 and October 2016, 44 interviews were conducted across the four countries included in the study. None of the designated interviewees at any of the sites refused to participate.

### Bangladesh

A total of 11 interviews were conducted in Bangladesh of which nine were with healthcare providers from the Chittagong Hill Tracts where the majority of the country’s malaria is reported [20], and two with policy makers.

#### Perception of malaria

Almost all respondents acknowledged that there had been a reduction of clinical malaria over the last two decades and attributed this to the effectiveness of implemented malaria control measures. Some respondents indicated that vivax malaria was no longer a public health priority.

*“8*-*10* *years back 70*-*80% of the hospitalized patients in Rangamati district were suffering from malaria. It was ranking first among the top ten diseases. Now it has changed a lot and we have controlled malaria significantly.''* Healthcare provider

*“No. I don’t see it [vivax malaria] as a problem here. Its proportion is very low.''* Healthcare provider

There were mixed perceptions regarding the seriousness of vivax malaria amongst the respondents. While most respondents regarded vivax to be a benign disease, others highlighted its propensity to recur and its potential social and economic impact.

*“Firstly it is a public health problem. Though vivax does not cause severe malaria as we all know, yet sufferers lose work hours, family earnings and health.''* Healthcare provider

All respondents stated that they were aware of the content of the malaria treatment guidelines and that they adhered to it. The main challenges for the treatment of vivax malaria were described as: (a) difficulty in correctly diagnosing the species of infection by microscopy and therefore concern that some patients might receive the wrong treatment; (b) access to appropriate treatment in remote areas; (c) adherence to a 14-day regimen of primaquine; (d) the usage of traditional medicine; and, (e) the lack of supply of anti-malarial drugs leading to stock outs in healthcare dispensaries.

*“Yes there are challenges. For example, in my working area there are many hard to reach areas as well as never to reach areas.''* Healthcare provider

*“Patients often don’t want to complete the course [of PQ*-*based radical cure] after improving. We have to insist a lot. Moreover, locals believe in traditional treatments even after getting well with our treatment. So we often say: ‘Go for your worshipping but complete the medications”* Healthcare provider

Respondents were aware of the risks of haemolysis after PQ treatment, however had not observed any cases in their daily practice.

*“It is known that haemolysis can happen in case of G6PD*-*deficient person, but I have not seen such [a] patient yet…”* Healthcare provider

Two interviewees stated that medical staff were often unaware about G6PD deficiency and that more training was needed.

*“Even when I train medical officers, I find that many are not aware of G6PD deficiency and its consequences. Not only … the public but [also] health professionals in this district don’t know enough about G6PD deficiencies in Rangamati. So, there is a certain gap of knowledge.”* Healthcare provider

#### Perceived need for G6PD testing

All respondent agreed that G6PD testing was not undertaken in patients with vivax malaria prior to prescribing PQ; however many mentioned that access to G6PD testing was desirable.

*“Yes it would be [useful]. Not only for malaria, but it will help [raise] the persons aware[ness] on certain drug intake and food restrictions also. If tests could be made available, then, we could also evaluate the affordability of the tests and it could be helpful before treating with primaquine.”* Healthcare provider

Cost and cost-effectiveness was stated by many respondents as the main barrier for the introduction of G6PD testing. Some thought that the test should be offered for free whereas others suggested a price in the range of 0.5–2.0 USD (40–150 BDT) per test.

*“As I have mentioned before, we are a highly populated and limited resourced country, so if the [G6PD] test could be made available in strip or RDT at less cost, then a lot of people can be benefitted.”* Healthcare provider

*“It [G6PD testing] should be free of cost like malaria RDT. People will not pay extra money to test G6PD when they are getting free [malaria] RDT and free treatment.”* Healthcare provider

A recurring theme when talking about the introduction of G6PD testing was the need for appropriate training of healthcare staff and concern regarding the laboratory infrastructure required to provide this.

#### Perceived knowledge gaps around G6PD

Most respondents indicated a knowledge gap on the local prevalence and severity of G6PD deficiency. Respondents also reported a need for better pharmacovigilance systems to monitor for severe haemolytic events.

*“I think there should be a study in these areas to know about G6PD deficiency and side effects of primaquine which we do not know. Then it will be possible to share it with the policy makers of the government.”* Healthcare provider

One respondent felt poorly informed about the G6PD tests available and their respective performance and operational characteristics.

### Cambodia

A total of 14 interviews were conducted in Cambodia, eight with policy makers and six with healthcare providers.

#### Perception of malaria

Malaria was recognized by all policy makers and healthcare providers as an important problem in the country. Healthcare providers stated that there had been a reduction in the number of cases presenting to clinics over the last 10 years.

*“Malaria is still a problem in this country but it seems to be dramatically decreased compared to the previous 10* *years ago.”* Policy maker

One respondent acknowledged that although falciparum cases had decreased while the proportion of vivax cases had increased.

*“Yes it’s a problem, because before [there was]… a lot of* Plasmodium falciparum *but right now* Plasmodium vivax *seems [to have] increased to 70% and* Plasmodium falciparum *[represents] only 20% [of malaria cases] and remaining are mixed [infections].”* Policy maker

Most respondents were aware that schizontocidal treatment was ineffective for treating the dormant liver stages of *P. vivax*. Although national treatment guidelines recommended PQ after testing for G6PD, it was not used routinely. Some respondents were unfamiliar with the current national treatment guidelines.

*“Primaquine was already there to be used to treat Pv [*P. vivax*] but the supplies… [have] not yet arrived, still in procurement process, so the NTG (treatment guidelines* – *the authors) …have not yet been fully implemented.”* Policy maker

*“Yes we often have relapses with malaria by* P. vivax*. It’s too difficult to settle with patient. We know* Plasmodium vivax *needs treatment by primaquine but don’t have any advice from the national programme. Patients lose faith in us.”* Healthcare provider

There was a general awareness of the risks of PQ use in G6PD-deficient patients; however, some healthcare workers expressed that they lacked information and knowledge on G6PD deficiency.

*“I don’t know [enough] as [I have] no guidance or training on G6PD.''* Healthcare provider

*“Never using G6PD test but have heard about this test.”* Healthcare provider

#### Perceived need for G6PD testing

National Cambodian guidelines recommend that PQ treatment only be administered after testing for G6PD deficiency for vivax radical cure. The introduction of routine G6PD testing was therefore considered desirable by both policy makers and healthcare providers. A number of respondents stated that routine testing would be useful in the context of malaria elimination.

*“For test[ing] G6PD[activity this] is very useful. I like G6PD test[ing] because when we use primaquine [the drug] can make blood damage, but if we do test before treatment and have problem with G6PD we can reduce [the] dose of primaquine.”* Policy maker

*“Very useful. If we have [a G6PD] test we expect [an improved]… treatment of vivax patients…better and join with malaria elimination.”* Healthcare provider

Respondents stated that the cost and cost effectiveness of routine G6PD testing was an important barrier to its introduction, as was the need for additional training of healthcare staff.

*“The price of [the] test should be subsidized…[so that] Cambodia can buy [the test].…The G6PD test must have price cheaper than price of malaria treatment.”* Policy maker

Two additional themes emerged when discussing the roll-out of routine G6PD testing: the need for community acceptance of G6PD testing and concerns regarding the feasibility of testing in the context of mass drug administration (MDA).

*“Providers, patients and communities must be well informed about the benefit of the G6PD test.”* Policy maker

*“For malaria elimination we need to apply MDA. So we discuss…the need to use test G6PD or not? … If [we] bring the test to rural areas [it] must [be] simple, easy to use.”* Policy maker

### China

In China, interviews were only conducted with policy makers. A total of six interviews were completed with policy makers from national, regional and technical levels.

#### Perception of malaria

All respondents acknowledged the Government’s efforts towards malaria elimination and stated that the burden of *P. vivax* had fallen but remained the predominant species of infection. Respondents mentioned the challenges in malaria control on endemic areas in the border regions.

*“Malaria now is only reported in some border areas and with some imported cases. Malaria local transmission is limited to the border area in Southern China since the National Malaria Elimination Campaign was launched in 2010, especially at the China*-*Myanmar border.”* Policy maker

All respondents stated that they were aware of the national treatment guidelines and that these were adhered to. Respondents were also aware of the risks of haemolysis after PQ treatment and that this could be severe in G6PD deficient individuals. Other significant challenges to successful treatment included patients’ adherence to a full course of treatment.

#### Perceived need for G6PD testing

Many respondents considered that the medical history was sufficient to decide whether to treat with PQ and that G6PD testing was therefore not undertaken routinely.

*“Currently, there is no compulsory test for G6PD before vivax treatment in the field, but vivax malaria patients will be asked whether they have a clinical history of broad bean haemolysis.”* Policy maker

The usefulness of G6PD testing was mainly mentioned in the context of research.

*“[G6PD testing] is conducted in some research study, especially in southern China, where G6PD deficiency was reported among minority groups.”* Policy maker

### Malaysia

In Malaysia a total of 13 interviews were conducted, four with policymakers and nine with healthcare providers from tertiary hospitals or outpatient clinics. Interviews were conducted in six states: Selangor, Pahang, Kelantan and Perak in Peninsula Malaysia, and Sabah and Sarawak in Borneo.

#### Perception of malaria

Interviewees acknowledged the reduced burden of malaria and its geographic distribution within Malaysia.

*“It [malaria] was [a problem]. But the problem is getting smaller. And now it’s localized to geographical regions and certain populations, at risk populations.”* Policy maker

*“It was a huge problem in the 1990s and I was working in a district. Oh no, it was in 1986…. and malaria was a big problem at that time. Along the way, things have improved*.” Policy maker

Many acknowledged a shift in the proportion of infections due to different species. The emergence of *P. knowlesi* was also stated as a major area of concern.

*“It shows that for vivax, the incidence is about the same, it actually went up a little for the last 20* *years. So the falciparum is the one coming down, so about 55*-*fold reduction in falciparum. So the one that actually overtaken falciparum and vivax is knowlesi. Knowlesi has overtaken both falciparum and vivax.”* Healthcare provider

*“Knowlesi no one knows how to control …but our malaria control is planned for other malaria but knowlesi, there is no game plan for knowlesi yet.”* Healthcare provider

Interviewees considered other infectious diseases more important than malaria in some parts of the country.

*“There is a significant proportion of malaria here [Borneo and Sabah] compared to the rest. In the Peninsula you have dengue being really, really big out there and it’s big over here but it’s the other way around for malaria. Dengue is a problem in Peninsula and malaria in Borneo.”* Healthcare provider

A recurring theme among nearly all of the interviewees was the challenge posed by imported cases due to foreign workers. Ensuring adequate treatment, follow-up and adherence for both indigenous patients and migrant workers was acknowledged as a major problem.

*“… even if you have like imported cases, they are very mobile. And some mobile populations. It’s very challenging to ensure that they are taking it [primaquine] for 14* *days. Either you need your staff to go there every day and like DOTS (directly observed treatment* – *the authors), you know, or you need to get [the mobile patients]. Get any other ways to know that how they take their medication for 14* *days.”* Policy maker

Malaria is a notifiable disease in Malaysia and patients diagnosed with malaria are followed up to ensure full recovery.

*“Actually we monitor every case. Every single case of malaria in Malaysia. And every P. vivax…, every case reported for malaria in the district level, it will be notified to a system called E*-*Notification. And then they do case investigation for this case and then our team in the district health office will do investigation and then they will register these patients into another system called E*-*VEKPRO (…) So, we look every details about patients management.''* Policy maker

#### Perceived usefulness of G6PD test

Respondents stated that G6PD testing by FST was performed routinely for vivax patients and within the national new-born screening programme. G6PD deficiency amongst the population living in malaria-endemic areas was perceived to be low:

*“G6PD deficiency is very…it’s quite rare, you’ll see less than one patient a year”* Healthcare provider

Despite perceptions of the low prevalence of G6PD deficiency, most respondents regarded the routine testing for G6PD to guide PQ treatment as convenient and acceptable.

*“We understand when to start the primaquine and avoid the adverse reactions, so its…I would say that it’s useful and its mandatory to know…”* Healthcare provider

Interviewees reported that the results of a newborn G6PD deficiency screening were recorded on a card and handed to the parent, nevertheless many felt that patients were often unaware of these results or had lost the recorded result. Hence most interviewees emphasized the need to redo G6PD testing in all vivax patients independent of earlier results or the availability of results from newborn screening:

*“We perform G6PD testing at birth and it is our practice to repeat the G6PD [test] prior to serving the primaquine. Once we know the patient have vivax and need primaquine…our G6PD test comes back within a day so there’s never been a delay of serving the primaquine because of G6PD [testing].”* Healthcare provider

One interviewee stated that he would administer PQ-based radical cure to his patients while waiting for a G6PD test result:

*“In fact, sometimes, when I see vivax patients I start primaquine right away. I just give it while waiting for test results, it won’t kill the patient. Nothing happens.”* Healthcare provider

#### Perceived need for better G6PD tests

The majority of respondents did not know which test was used in routine practice, however some correctly mentioned the FST and considered its operational characteristics to be favourable:

*“It’s a very simple, fast test. I can get the results on the same day. It doesn’t sound very complicated to me. It’s easily available.”* Healthcare provider

*“It’s convenient, it does the job…it does the job of finding out for me who is G6PD [deficient]…that’s it.”* Healthcare provider

While most respondents did not perceive an urgent need for improved G6PD tests, some raised concerns over the sensitivity and specificity of the FST.

*“The qualitative test comes with an element of false positives and false negatives, and come in as equivocal.”* Healthcare provider

One provider raised the issue about objectiveness in the reading of the FST.

*“I used to be quite unhappy with the G6PD screening test because there are too many positives and it’s based on just the fluorescent spots so the interpretation is very subjective so that’s my main problem with the G6PD test.”* Healthcare provider

Some mentioned the benefits of spectrophotometry or flow cytometry as a diagnostic tool that provides quantitative results rather than qualitative results, but also acknowledged that for most patients this might not be needed.

*“I guess all tests have their limitations so certain conditions where the heterozygous females, people worry you might [be] miss[ing] by the screening test.”* Healthcare provider

After describing the benefits of a RDT, some interviewees suggested that a respective assay would be helpful for patient management in remote regions.

*“Yes, yes in the field [an RDT could be useful]. In places which are further away from the major hospitals or health clinics, then yes. In the tertiary setting, I think the information we have is good enough.”* Healthcare provider

In general there was a consensus that in the current situation and with regards to cost constraints, there was no immediate need for the introduction of point-of-care tests for routine settings.

*“…there are a lot of point*-*of*-*care tests but you have to evaluate how the point*-*of*-*care test will add value to management and also consider cost effectiveness. So if its life saving, then sure but for the ease of patient coming back for results, of course it will be an added benefit for point*-*of*-*care test but most of our malaria patients are in*-*patient so they won’t be walking around. I mean if I had unlimited resources, a point*-*of*-*care test would be great but if I had to choose, a G6PD point*-*of*-*care test will not be the first thing to come to my mind.”* Healthcare provider.

## Discussion

This study aimed to understand the perceived need for routine G6PD testing prior to PQ-based radical cure and identify key barriers to the introduction of routine G6PD testing.

In Bangladesh and China, PQ treatment is provided without prior G6PD testing [21]. In contrast, Cambodia has G6PD testing prior to PQ administration included in the national guidelines, but neither testing nor treatment is currently provided on a routine basis [17, 21]. Malaysia, where PQ is provided on a routine basis to vivax patients following G6PD testing [18], was included in the study as a comparator and to understand satisfaction with the currently used test format and how satisfied health care providers were with the current testing procedures.

In Bangladesh, Cambodia and China three key barriers impeding the introduction of routine G6PD testing prior to PQ based radical cure were identified.

### 1. Perceived low risk of primaquine-induced haemolysis

The risk of PQ-induced haemolysis was considered to be low and this was a significant factor for the perceived need to test a patients G6PD status; treatment providers from Bangladesh were not aware of any case with PQ induced haemolysis. There are several plausible explanations for the perception of low haemolytic risk of PQ treatment. In remote areas patients may not be able to return to the healthcare facility despite significant drug-induced haemolysis, and thus significant haemolysis may have gone undetected. Alternatively poor adherence to treatment [22–25] could have resulted in patients receiving only low doses of PQ with minimal haemolytic risk. If the prevalence of G6PD deficiency was very low, or the local variant conferred only a mild and self-limiting haemolysis then patients with clinically significant haemolysis would be rare or unlikely to seek further medical attention. Alternatively, the protective effect of G6PD deficiency against a *P. vivax* infection could be sufficiently high to result in low numbers of patients with G6PD deficiency being exposed to drug-induced haemolysis. In Malaysia the prevalence of G6PD deficiency was considered low, however healthcare providers still adhered to the national testing algorithm. Indeed contrary to the other countries, the G6PD status of a patient was considered “mandatory to know”, with much stricter adherence to the national guidelines.

### 2. *Plasmodium vivax* is considered “benign” or of very low incidence

In general, respondents regarded *P. vivax* infections as benign and/or of low incidence, whereas other communicable and non-communicable diseases were regarded as higher priority. In Bangladesh, Cambodia and China the declining incidence of *P. vivax* highlighted the success of national malaria programmes, but such success may have paradoxically undermined elimination efforts due to reduced awareness of the disease and subsequent funding cuts. This paradox has been proposed as a key reason why the first Global Malaria Eradication Programme (GMEP) ultimately failed [26]. However in Malaysia where there has been a major decrease in the reported burden of disease, the elimination path has not been impeded. Patients diagnosed with malaria are admitted to hospital until schizontocidal treatment is completed. All respondents from Malaysia were aware of the challenges of elimination settings, the threat of imported malaria and the need for sustained public control efforts.

### 3. Routine G6PD testing generates additional costs

The perceived low prevalence of G6PD deficiency and reduced incidence of *P. vivax* infection lead many respondents to question the cost-effectiveness of routine G6PD testing. In countries with limited public health resources this issue is critical for the implementation of routine testing. Regional or country-specific cost-benefit analyses [27], such as one recently conducted in Brazil [28] will be key to encouraging policymakers to invest adequately in routine G6PD testing. Respective economic studies are currently underway in a number of countries in Asia [29]. A recent analysis of data gathered from the Thai–Myanmar border suggested that routine testing had the potential to reduce total healthcare costs [30].

In Malaysia FST testing prior to PQ treatment is recommended and newborn babies are routinely screened for G6PD deficiency. Respondents believed that the current practice of admitting all patients diagnosed with malaria to hospital for treatment was an effective strategy. Only providing PQ once a patients’ G6PD status was known was considered practical and feasible.

Although newborn infants are screened for G6PD deficiency in Malaysia, most interviewees emphasized that those results are rarely referred to, since patients cards are often missing or unavailable for older patients born before the introduction of newborn screening. Furthermore, G6PD enzyme activity may be elevated in early life and this may mask G6PD deficiency. Re-testing of patients irrespective of the result of the newborn screening is therefore beneficial to patients [31].

Some interviewees highlighted that a limitation of the FST test was its subjective interpretation and low performance. While currently available RDTs for G6PD have comparable performance to the FST [16, 32], their operational characteristics are more favourable to the FST. Most currently available G6PD RDTs do not require a cold chain or laboratory infrastructure [33, 34], and can be undertaken at the bed-side on an individual basis (point-of-care). In contrast, the FST requires laboratory facilities and is generally run in batches for multiple patients; as the incidence of malaria falls, such batch testing will no longer be economical. Although test results for RDTs are available at the bedside, most Malaysian respondents did not see a particular benefit in replacing the currently used FST by a RDT. The advantage of instant test results could be more relevant for marginalized groups, including those living in remote areas or for migrant workers with limited access to the health system where admission to hospitals is difficult and follow-up is limited. The benefits of rapid results were also mentioned by Cambodian respondents in the context of MDA, a setting where instant results are needed and easy-to-use tests warranted.

The study has a number of limitations. Firstly participants were selected through convenient sampling, an approach which was justified by the small number of interviewees and the specific technical knowledge required. Furthermore summaries of all interviews were sent back to interviewees for approval which was agreed during the approval for the study. However in no case were the summaries altered. Secondly the proportions of policy decision makers and healthcare providers varied from country to country and a variety of interview methods were deployed including in-person, by phone, in English or the local language. Whilst these may have influenced the comparison of data between countries, they were consistent within country and are unlikely to have affected the main barriers identified. Thirdly whilst our findings are limited to four Asian countries the key barriers identified are likely to be generalizable to other locations in vivax endemic countries although further studies are warranted to confirm this.

In conclusion the findings of the study highlight the need for greater awareness of the importance of *P. vivax* radical cure and the risks of PQ induced haemolysis. Cost-effectiveness and cost-benefit studies on the introduction of routine G6PD testing in *P. vivax*-endemic settings, with complementary information on the local prevalence and genetic variants of G6PD deficiency will be critical in helping policymakers to prioritize limited resources. Strengthening pharmacovigilance systems to monitor the haemolytic risks associated with primaquine will also prepare malaria control programmes for the introduction of other 8-aminoquinolone based treatment options with easier treatment schedules and the enhanced malaria control strategies needed to achieve the elimination of *P. vivax*.

## Additional files

**Additional file 1.** English version of the interview guide for healthcare deliverers.

**Additional file 2.** English version of the interview guide for policy decision makers.
