# Supplementary material for: Barriers to routine G6PD testing prior to treatment with primaquine
Source: Malar J. 2017 Aug 10;16:329. doi: 10.1186/s12936-017-1981-y (PMC5553859; doi:10.1186/s12936-017-1981-y)
Supplement: Supplementary file 1 — Additional file 1. English version of the interview guide for healthcare deliverers. [file 12936_2017_1981_MOESM1_ESM.docx]

Additional file 1: English version of the interview guide for healthcare deliverers:

Respondent ID:  -    -   -

Team Number letter Location Office

Interviewer Initials: _____________ Note Taker Initials: _____________

Date of Interview: ____ /____ /2015 (mo/dy/yr)

Start Time: ____:____ AM/PM End Time: ____:____ AM/PM

Recording number: ________________________________________________

**TURN ON THE RECORDER**

Introduction and Purpose

Hello my name is [SPECIFY NAME] from [SPECIFY ORGANIZATION]. The University of California, San Francisco, USA, the Menzies School of Health Research, Darwin, Australia, the Asia Pacific Malaria Elimination Network, and the US Centers for Disease Control and Prevention are conducting an assessment to learn about the treatment of malaria and associated barriers in delivering safe vivax malaria treatment. This assessment will help us understand how people’s malaria treatment can be improved. You have been recommended as a person directly involved in health decisions regarding malaria in your country and we would like to ask you to take part in an interview about malaria treatment today. If you do not want to take part you can simply tell me without having to provide any reasons for your decision. If you agree to the interview and later on change your mind, you can let me know at any point in time throughout the interview and I will delete any information already collected and under no circumstances will you have to face negative consequences from your decision.

Procedures and Consent

To facilitate our note-taking, we would like to audio tape our conversations today. For your information, only researchers on the project will have access to the tapes which will be destroyed at study end. By agreeing to participate in this interview you are giving your oral consent to participate in this study. By agreeing to the interview you understand that: (1) all information will be held confidential to the extent allowed by law, (2) your participation is voluntary and you may stop at any time if you feel uncomfortable, (3) we do not intend to inflict any harm, and (4) you grant us permission to use data and extract information from this interview and publish in a format that will not allow to identify you as a person. Thank you for agreeing to participate, you will not face any consequences if you decide to withdraw your consent at any time point during the interview.

Persons to Contact

If, at any time, you have questions related to this assessment, you may contact [INSERT APPROPRIATE CONTACT]. If you have questions or concerns about your rights as a research participant, you can call the [national board that looks into these things].

Thank you for [AGREEING TO PARTICIPATE] or [YOUR TIME].

1. **BACKGROUND**
2. Please tell me about the specific roles and responsibilities in your job? **PROBE IF NOT DESCRIBED:** When did you take this job? How long have you been working in the field of malaria? What were you doing before you took this job?
3. What is your educational and training background? (check that a-d is answered)
   1. What is your highest degree?
   2. What is your current position?
   3. How long have you been in your current position?
   4. Where have you worked before this job? **PROBE IF NOT DESCRIBED:** When did you take this job? How long have you been working in the field of malaria? What were you doing before you took this job?
4. **MALARIA AND SERIOUSNESS OF VIVAX MALARIA**
5. Do many of the patients you see suffer from malaria?
   1. Is vivax malaria a problem in your country? *[If the respondent does not know what vivax malaria is, please define “vivax malaria is one of four types of malaria that generally causes a milder form of malaria”]*.
   2. On average, what proportion of malaria cases has vivax malaria in your country?
6. **CASE MANAGEMENT FOR VIVAX MALARIA**
7. How are you treating vivax malaria in the field currently?
8. What are the national treatment guidelines?
9. Are there any differences between the national guidelines and what really happens in the field?
10. If yes: What is the reason for this difference?What are the challenges with the treatment you provide?
11. **PRIMAQUINE TREATMENT**
12. What problems are associated with primaquine treatment in vivax malaria?
13. **G6PD TESTING PRACTICES AND GUIDELINES**
14. Do you perform G6PD testing prior to primaquine treatment? *[If the respondent does not know the meaning of G6PD, please define: “glucose-6-phosphate dehydrogenase is an enzyme. Some individuals have reduced levels of this enzyme which affect their ability to process certain medicines, such as primaquine”]*.
    1. If yes, what test is used?
    2. Do you find the test useful?
       1. If yes, what in particular do you like about the test that is currently in use?
       2. If no, what in particular do you dislike about the test that is currently in use?
       3. Are there ways in which the test could be improved?
    3. When do you perform G6PD testing?
15. **ECONOMICAL ASPECTS OF G6PD TESTING**
16. Where do you get your supply of G6PD tests?
17. Do you have a constant and sufficient supply of G6PD tests at your health facility?
18. If not why not?
19. What are some of the challenges of receiving a constant and sufficient supply of G6PD tests?
    1. How do these challenges affect the conduct of malaria case management activities?
    2. How do you address these challenges?
    3. What needs to be done to eliminate these challenges?
20. What is the price for one G6PD test at the moment in your setting?
    1. Do you consider this price appropriate?
    2. Can people afford it?
    3. How often is the test not conducted because they cannot afford to buy it?
    4. If people cannot afford the test, do you think (current price minus 50 US Cents) would be an affordable price? (*Continue reducing the price by 50 USCents until the respondent believes the price is affordable)*
21. **PERCEIVED KNOWLEDGE GAPS ON G6PD TESTING**
22. What information (if any) are you missing on G6PD and G6PD testing?
    1. Do you know whom to contact to find out the missing information?
23. **FINAL COMMENTS OR SUGGESTIONS**
24. Our key interest for this interview was to understand key barriers to routine G6PD testing prior to primaquine treatment. Would you like to add anything to this topic?
25. **CLOSING**
26. This is the end of my questions. Given some of the things that we talked about today, are there other key informants I should speak to who could tell me more about these topics?
27. Thank you so much for your time. Do you have any questions for me?
