# Supplementary material for: Barriers to routine G6PD testing prior to treatment with primaquine
Source: Malar J. 2017 Aug 10;16:329. doi: 10.1186/s12936-017-1981-y (PMC5553859; doi:10.1186/s12936-017-1981-y)
Supplement: Supplementary file 2 — Additional file 2. English version of the interview guide for policy decision makers. [file 12936_2017_1981_MOESM2_ESM.docx]

Additional file 2: English version of the interview guide for policy decision makers:

**KEY INFORMANT interview guide**

Respondent ID:  -    -   -

Team Number letter Location Office

Interviewer Initials: _____________ Note Taker Initials: _____________

Date of Interview: ____ /____ /2015 (DD/MMM)

Start Time: ____:____ AM/PM End Time: ____:____ AM/PM

Recording number: ________________________________________________

**TURN ON THE RECORDER**

Introduction and Purpose

Hello my name is [SPECIFY NAME] from [SPECIFY ORGANIZATION]. The University of California, San Francisco, USA, the Menzies School of Health Research, Darwin, Australia, the Asia Pacific Malaria Elimination Network, and the US Centers for Disease Control and Prevention are conducting an assessment to learn about the treatment of malaria and associated barriers in delivering safe vivax malaria treatment. This assessment will help us understand how people’s malaria treatment can be improved. You have been recommended as a person directly involved in health decisions regarding malaria in your country and we would like to ask you to take part in an interview about malaria treatment today. If you do not want to take part you can simply tell me without having to provide any reasons for your decision. If you agree to the interview and later on change your mind, you can let me know at any point in time throughout the interview and I will delete any information already collected and under no circumstances will you have to face negative consequences from your decision.

Procedures and Consent

To facilitate our note-taking, we would like to audio tape our conversations today. For your information, only researchers on the project will have access to the tapes which will be destroyed at study end. By agreeing to participate in this interview you are giving your oral consent to participate in this study. By agreeing to the interview you understand that: (1) all information will be held confidential to the extent allowed by law, (2) your participation is voluntary and you may stop at any time if you feel uncomfortable, (3) we do not intend to inflict any harm, and (4) you grant us permission to use data and extract information from this interview and publish in a format that will not allow to identify you as a person. Thank you for agreeing to participate, you will not face any consequences if you decide to withdraw your consent at any time point during the interview.

Persons to Contact

If, at any time, you have questions related to this assessment, you may contact [INSERT APPROPRIATE CONTACT]. If you have questions or concerns about your rights as a research participant, you can call the [national board that looks into these things].

Thank you for [AGREEING TO PARTICIPATE] or [YOUR TIME].

| 1. **BACKGROUND** 2. First, can you help us to understand what the roles and responsibilities of your office are? (check that a,b is answered)    1. What types of activities is your office involved in?    2. Where does malaria currently fit into those activities? 3. Please tell me about your specific roles and responsibilities in your job? **PROBE IF NOT DESCRIBED:** When did you take this job? How long have you been working in the field of malaria? What were you doing before you took this job? 4. What is your educational and training background? (check that a-d is answered)    1. What is your highest degree?    2. What is your current position?    3. How long have you been in your current position?    4. Where have you worked before this job? **PROBE IF NOT DESCRIBED:** When did you take this job? How long have you been working in the field of malaria? What were you doing before you took this job? 5. Who in your country makes decisions on national guidelines for malaria treatment? 6. How are these decisions made? **PROBE IF NOT DESCRIBED:** Who is consulted? What information do you use to help you make the decision? 7. **MALARIA AND SERIOUSNESS OF VIVAX MALARIA** 8. Is malaria a common health problem in your country? 9. Is vivax malaria a problem in your country? *[If the respondent does not know what vivax malaria is, please define “vivax malaria is one of four types of malaria, that generally causes a milder form of malaria”]*. 10. On average, what proportion of malaria cases has vivax malaria in your country? 11. **CASE MANAGEMENT FOR VIVAX MALARIA** 12. What are the current treatment guidelines for vivax malaria? 13. And what is standard practice in the field?     1. If there is a difference between national guidelines and practiced vivax malaria treatment, why do you think this is the case? 14. **PRIMAQUINE TREATMENT** 15. What problems are associated with primaquine treatment in vivax malaria? 16. **G6PD TESTING PRACTICES AND GUIDELINES** 17. Do the national guidelines recommend performing glucose-6-phospohate dehydrogenase (G6PD) tests prior to primaquine treatment? *[If the respondent does not know the meaning of G6PD, please define: “glucose-6-phosphate dehydrogenase is an enzyme vital for red blood cells. Many people have poorly functioning enzyme, they are G6PD deficient. Under every day conditions this does not affect the health and general well being of the individual. However some compounds, such as primaquine can cause a rapid disintegration of red blood cells and can trigger very severe conditions in G6PD deficient individuals called haemolysis if treated with primaquine”]*.     1. If yes, what test is used?     2. If yes, are the tests procured and distributed and how?     3. Who administers them?     4. Do you find the test useful?        1. If yes, what in particular do you like about the test that is currently in use?        2. If no, what in particular do you dislike about the test that is currently in use?        3. Are there ways in which the test could be improved?      1. **ECONOMICAL ASPECTS OF G6PD TESTING** 2. Do you have regular access to the funds you need to procure G6PD tests? 3. What is the procurement cost for one test? 4. What are some of the challenges of procuring and delivering G6PD tests?    1. How do these challenges affect the conduct of malaria case management activities?    2. What suggestions or strategies do you have for improving this situation? 5. What is the price for one G6PD test at the moment in your setting? **PROBE IF NOT DESCRIBED:** What is the cost of procuring the test? How much are the tests being sold for (if sold)?    1. If sold:       1. Do you consider this price appropriate?       2. If not, what do you think would be an affordable price?       3. For how much do you buy the test? 6. **PERCEIVED KNOWLEDGE GAPS ON G6PD TESTING** 7. What information (if any) are you missing on G6PD and G6PD testing?    1. Do you know whom to contact to find out if the missing information exists? 8. **FINAL COMMENTS OR SUGGESTIONS** 9. Our key interest for this interview was to understand key barriers to routine G6PD testing prior to primaquine treatment. Would you like to add anything to this topic? 10. **CLOSING** 11. This is the end of my questions. Given some of the things that we talked about today, are there other key informants I should speak to who could tell me more about these topics? 12. Thank you so much for your time. Do you have any questions for me? |
| --- |
